# *Citrus unshiu* peel extract alleviates cancer-induced weight loss in mice bearing CT-26 adenocarcinoma

**DOI:** 10.1038/srep24214

**Published:** 2016-04-11

**Authors:** Aeyung Kim, Minju Im, Min Jung Gu, Jin Yeul Ma

**Affiliations:** 1 Korean Medicine (KM) Application Center, Korea Institute of Oriental Medicine (KIOM), 70 Chumdan-ro, Dong-gu, Daegu 701-300, Republic of Korea

## Abstract

Skeletal muscle atrophy is a critical feature of cancer-induced cachexia, caused by pro-cachectic factors secreted by host cells and tumor cells. Therefore, blockade of these factors has considered a reasonable target for pharmacological and nutritional interventions to prevent skeletal muscle loss under cancer-induced cachexia. *Citrus unshiu* peel (CUP) has been used for treating the common cold, dyspepsia, and bronchial discomfort and reported to have pharmacological activities against inflammation, allergy, diabetes, and viral infection. In the present study, we observed that daily oral administration of water extract of CUP (WCUP) to male BALB/c mice bearing CT-26 adenocarcinoma remarkably reduced the losses in final body weight, carcass weight, gastrocnemius muscle, epididymal adipose tissue, and hemoglobin (Hb), compared with saline treatment. The levels of serum IL-6 and muscle-specific E3 ligases elevated by tumor burden were also considerably reduced by WCUP administration. In an *in vitro* experiment, WCUP efficiently suppressed the production of pro-cachectic cytokines in immune cells as well as cancer cells. In addition, WCUP treatment attenuated C2C12 skeletal muscle cell atrophy caused by cancer cells. These findings collectively suggest that WCUP is beneficial as a nutritional supplement for the management of cancer patients with severe weight loss.

Cachexia is defined as a complex metabolic syndrome closely associated with many illnesses and characterized by a loss of muscle, with or without a loss of fat mass, followed by a progressive loss of body weight. These wasting conditions are clearly distinct from starvation and age-related muscle loss, so they cannot be completely prevented or reversed by nutritional support. Cachexia occurs in some types of cancer, chronic heart failure, chronic obstructive pulmonary disease, streptozotocin-induced diabetes, and uremia, decreases the length and quality of life (QoL), and leads to progressive functional impairment^1^,^2^,^3^. In cancer patients, cachectic conditions including anorexia, fatigue, metabolic and endocrine alterations, and loss of lean body mass negatively impact the responses to conventional chemotherapy and radiotherapy, QoL, and mortality and morbidity rates. More than 50% of cancer patients suffer from cachectic symptoms, and up to 20% of patients die directly due to cancer-induced cachexia. Weight loss is more frequently experienced by patients with gastric and pancreatic cancer than by those with breast cancer, sarcomas, and leukemia^4^,^5^.

Although the molecular basis of cancer cachexia has not been resolved completely, pro-inflammatory cytokines, including TNF-α, IL-1β, IL-6, and IFN-γ, produced by tumor cells themselves or host immune cells in response to tumors have been identified to play key roles as the main humoral factors responsible for muscle wasting^6^,^7^,^8^. TNF-α suppresses myogenic differentiation through NF-κB activation and promotes skeletal muscle degradation by inducing E3 ligases such as muscle atrophy F-box (MAFbx) and muscle-specific RING finger protein (MuRF1). Circulating IL-6 levels have been closely associated with weight loss and reduced survival in cancer patients. In addition, tumor-specific factors such as lipid-mobilizing factor (LMF) and proteolysis-inducing factor (PIF) are capable of degrading adipose tissue and skeletal muscle, respectively^8^,^9^,^10^. In this regard, treatment with anti-cytokine antibodies, cytokine receptor agonists, or myostatin (Mstn) blockade using activin IIB receptor (actRIIB) antagonists reduced muscle wasting and improved physical performance in the tumor-bearing experimental cachexia model^8^,^11^,^12^,^13^. In several cases, anti-cytokine therapies have been shown to be effective in reversing cancer-induced cachexia; however, the routine use of antibodies is unfeasible due to the high cost and lack of clinical data.

Several medical trials on the treatment of cancer cachexia have been performed using synthetic drugs that can stimulate appetite, suppress systemic inflammation, and inhibit tumor-induced protein degradation; however, they are limited due to the unwanted side effects and low *in vivo* efficacy. Among them, medroxyprogesterone acetate (MPA) has been widely used as a supportive therapy for patients with cancer- or acquired immune deficiency syndrome-related cachexia, acting as an appetite stimulant. However, the long-term use of MPA has toxic effects, including diabetes, mood swings, osteoporosis, and thromboembolism^14^,^15^. In addition, eicosapentaenoic acid, thalidomide, and nonsteroidal anti-inflammatory drugs also induce adverse effects such as nausea, gastrointestinal upset, and hemorrhage^16^,^17^. Therefore, finding novel agents that efficiently prevent and/or treat cancer-induced cachexia with minimal adverse effects is very important to improve the prognosis of cancer therapy, the survival time, and QoL of cancer patients.

Recently, herbal medicines have received increasing attention as attractive adjuvants for the management of intractable diseases such as cancer in terms of their high efficacy and minimal toxicity. The combination of *Scutellaria baicalensis* and Qing-Shu-Yi-Qi-Tang, a multi-component herbal extract, effectively ameliorated the cachectic symptoms caused by chemotherapy and improved therapeutic efficacy^18^. The Kampo formula Hochuekkito (TJ-41) and the natural herb *Coptidis rhizoma* improved cachexia symptoms in mice induced by Colone-26 adenocarcinoma via a reduction in IL-6 levels^19^.

*Citrus unshiu* Markov. is a seedless and easily peeled citrus fruit, the dried peel of which has been widely used in Korea, China, and Japan for medicinal purposes to improve bronchial conditions and blood circulation and to treat vomiting and dyspepsia. In addition, *Citrus unshiu* peel (CUP) has been reported to possess potent anti-inflammatory, anti-oxidant, anti-diabetic, anti-bacterial, and anti-allergic activities^20^,^21^,^22^. However, studies on the anti-cachectic effect of CUP and its underlying mechanisms have not been performed.

In the present study, we aimed to investigate whether water extract of CUP (WCUP) alleviates cancer-induced cachexia symptoms in mice bearing CT-26 adenocarcinoma, including losses in body weight, skeletal muscle, and fat mass. In addition, using the J774A.1 murine macrophage cell line and the C2C12 murine myoblast cell line, the effects and *in vitro* mechanism of WCUP on the production of pro-cachectic cytokines and muscle atrophy were investigated in detail.

## Results

### WCUP treatment in CT-26 tumor-bearing mice attenuates cancer-induced weight loss and cachexia symptoms

**Figure 1:**
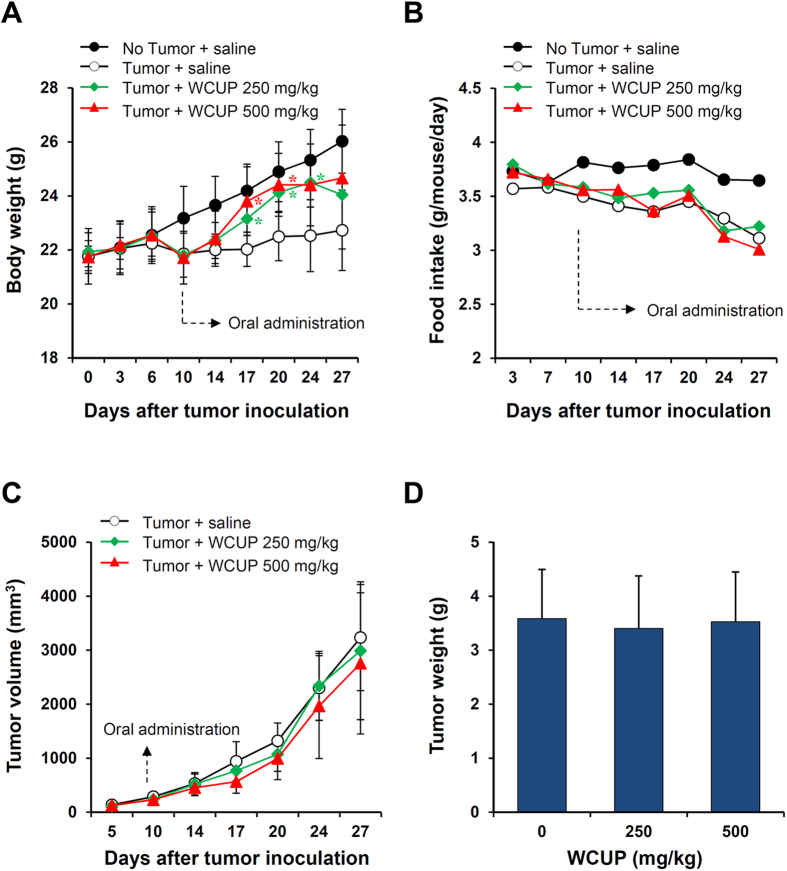
Effects of WCUP on body weight, tumor growth, and food intake in CT-26 tumor-bearing mice. Male BALB/c mice (n = 15) were subcutaneously injected with CT-26 cells in the abdominal region. On day 10 after tumor inoculation, the mice were divided randomly into three groups (n = 5 per group) and administered WCUP daily at doses of 250 and 500 mg/kg, or saline for 17 days. Control mice with no tumors (n = 5) were also administered an equal volume of saline daily during the experiment. Body weight **(A)**, food intake **(B)**, and tumor volume **(C)** were compared on days 14, 17, 20, 24, and 27. At the time of sacrifice, tumors were removed and weighed **(D)**. Animal experiments were repeated three times, and representative results are shown. Data are presented as means ± SD. Statistical significance was determined with Student *t*-test. *p < 0.05, significantly different from the group of ‘tumor + saline’ in the same day (on day 17; tumor + WCUP 250 mg/kg, *p* = 0.0210, tumor + WCUP 500 mg/kg, *p* = 0.0234) (on day 20; tumor + WCUP 250 mg/kg, *p* = 0.0123, tumor + WCUP 500 mg/kg, *p* = 0.0193) (on day 24; tumor + WCUP 250 mg/kg, *p* = 0.0258).

**Figure 2:**
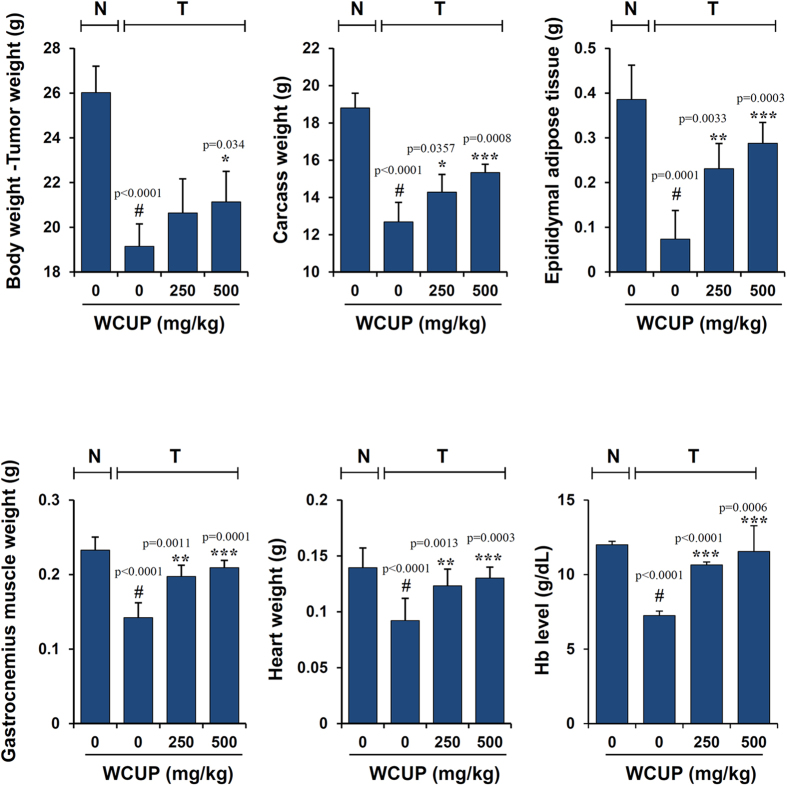
Effects of WCUP on cachectic parameters in CT-26 tumor-bearing mice. After sacrifice on day 27, the carcasses, epididymal adipose tissues, gastrocnemius muscles, and hearts of the mice were weighed. Hb levels in blood were determined using the ADVIA 2120i hematology system (n = 5 per group). Data are representative of three independent experiments and expressed as means ± SD. Statistical significance was evaluated with Student *t*-test. Significantly different from the group of normal mice with no tumor; ^#^*p* < 0.001. Significantly different from the group of tumor + saline; *p < 0.05, **p < 0.01, ***p < 0.001. N, normal mice with no tumor; T, tumor-bearing mice.

Cancer cachexia is a complex syndrome characterized by anorexia, loss of skeletal muscle and adipose tissue, and involuntary weight loss despite a nutritional supply, and it is closely correlated with high mortality and poor prognosis in cancer patients^17^. To examine whether WCUP is effective against cancer-induced cachexia, CT-26 tumor-bearing mice showing reductions in body weight and food intake were orally administered WCUP on a daily basis and monitored. Body weight increased steadily in normal healthy mice but not in CT-26 tumor-bearing mice, resulting in a difference in body weight of approximately 10% regardless of tumor weight on day 10 after tumor injection (Fig. 1A). Over the course of the whole experiment lasting 27 days, normal mice increased their body weight by 19.58%, while saline-treated control mice increased it by 4.47%. Mice treated with WCUP at doses of 250 and 500 mg/kg increased their body weight by 9.62% and 13.46%, showing recovery of their weights to approximately 92.38% and 94.76% of that of normal mice, respectively. The average food intakes/mouse/day during the entire experimental period for normal, control, and 250 mg/kg WCUP-treated and 500 mg/kg WCUP-treated mice were 3.74 ± 0.08, 3.33 ± 0.16, 3.39 ± 0.20, and 3.31 ± 0.21, respectively, indicating that tumor burden induces a loss of appetite, and WCUP treatment does not improve appetite in CT-26 tumor-bearing mice (Fig. 1B). Tumor growth was not suppressed by WCUP administration, and tumor weights were similar among control, 250 mg/kg WCUP-treated, and 500 mg/kg WCUP-treated mice at 3.58 ± 0.91, 3.41 ± 0.97, and 3.53 ± 0.92, respectively (Fig. 1C,D). At the time of sacrifice, significant reductions in final body weight (p < 0.0001), carcass weight (p < 0.0001), epididymal adipose tissue (p = 0.0001), gastrocnemius muscle (p < 0.0001), and heart weight (p < 0.0001) were observed in the control mice compared with normal mice. In addition, Hb levels in control mice were also decreased by approximately 40% compared with the levels in normal mice (p < 0.0001). The oral administration of WCUP at doses of 250 and 500 mg/kg for 17 consecutive days starting on day 10 significantly prevented the losses in final body weight (F = 3.086, p = 0.0829), carcass weight (F = 12.11, p = 0.0013), and heart weight (F = 17.05, p = 0.0003), as well as the wasting of epididymal adipose tissue (F = 19.63, p = 0.0002) and gastrocnemius muscle (F = 26.80, p < 0.0001), in CT-26 tumor-bearing mice compared with saline-treated control mice. Furthermore, Hb levels in WCUP-treated mice were maintained at levels similar to those in normal mice (F = 24.86, p < 0.0001) (Figs 2 and S1). These results indicate that WCUP administration is helpful to maintain body weight in tumor-bearing mice and to ameliorate cachexia symptoms, with no influence on appetite or tumor growth.

### WCUP treatment in CT-26 tumor-bearing mice decreased serum IL-6 levels and muscle degradation-related protein expression

**Figure 3:**
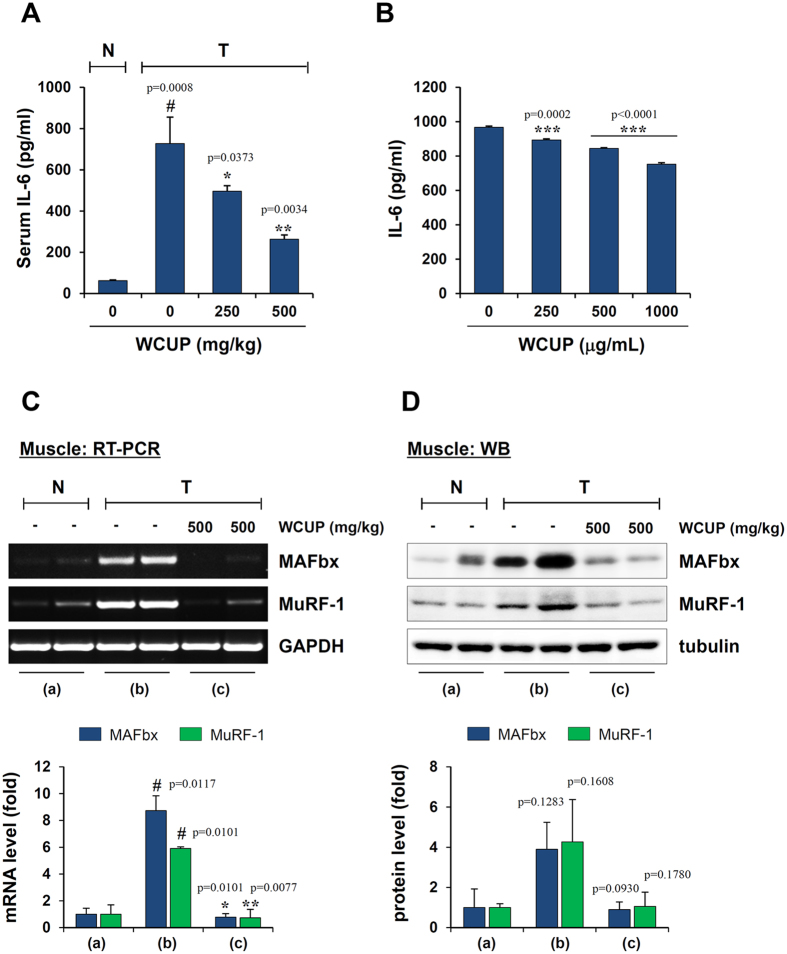
Effect of WCUP on levels of serum IL-6 and muscle atrophy-related proteins in CT-26 tumor-bearing mice. **(A)** After sacrifice, serum IL-6 levels in mice were determined by ELISA (n = 3 per group). Statistical significance was evaluated with Student *t*-test. ^#^*p* < 0.001 vs. normal mice with no tumor, *p < 0.05 and **p < 0.01 vs tumor + saline. **(B)** CT-26 cells were treated with the indicated concentrations of WCUP for 48 h, and then the IL-6 levels in culture supernatants were measured by ELISA (n = 3 per group). Statistical significance was evaluated with Student *t*-test. ***p < 0.001 vs. WCUP-untreated control cells. **(C,D)** mRNA and protein levels of MAFbx and MuRF-1 in gastrocnemius muscle were examined by RT-PCR and Western blotting, respectively (n = 2 per group). The levels of GAPDH and tubulin were used for normalization of the samples. Bar graph presents the means ± SD. Statistical significance was evaluated with Student *t*-test. ^#^*p* < 0.05 vs. normal mice with no tumor, *p < 0.05 and **p < 0.01 vs tumor + saline. The full size gels and blots were shown in the Supplementary Fig. S3 and band of interest is indicated with an arrow.

To further elucidate the mechanisms underlying the anti-cachectic effect of WCUP, we measured serum IL-6 levels in mice. Tumor burden caused a marked increase in the serum IL-6 level (p = 0.0008), and the administration of WCUP significantly decreased the IL-6 level in the serum of CT-26 tumor-bearing mice in a dose-dependent manner (F = 27.73, p = 0.0009) (Fig. 3A). It has been reported that, in cachexia, tumor-derived inflammatory cytokines such as IL-6, TNF-α, and IL-1 are critical inducers of muscle wasting and fat depletion^6^. To examine whether WCUP can reduce the production of tumor-derived cytokines, CT-26 cells were treated with 250, 500, and 1000 μg/mL WCUP, and then the levels of mRNA and IL-6 secretion were determined. WCUP up to 1000 μg/mL was non-cytotoxic and significantly reduced the level of IL-6 mRNA (Fig. S2). In addition, WCUP treatment suppressed the IL-6 production in CT-26 cells in a dose-dependent manner (F = 583.1, p = 0.0001) (Fig. 3B). The levels of TNF-α and IL-1β in the serum of mice and culture supernatants of CT-26 cells were below the detection limit in all groups (data not shown). To further characterize the cachexia induced by the tumor, the expression of the muscle-specific ubiquitin ligases MAFbx and MuRF-1 was assessed in gastrocnemius muscles. As reported previously^23^, MAFbx and MuRF-1 mRNA expression was dramatically elevated in the muscles of CT-26 tumor-bearing mice compared with normal mice (Fig. S2). Consistent with its preventive effect on muscle wasting, WCUP administration reduced the levels of MAFbx and MuRF-1 mRNA and protein in the muscles of CT-26 tumor-bearing mice (Fig. 3C,D). These findings indicate that WCUP efficiently suppresses CT-26 tumor-induced inflammatory responses and delays muscle breakdown.

### WCUP downregulates LPS-induced inflammatory cytokine production in J774A.1 cells

**Figure 4:**
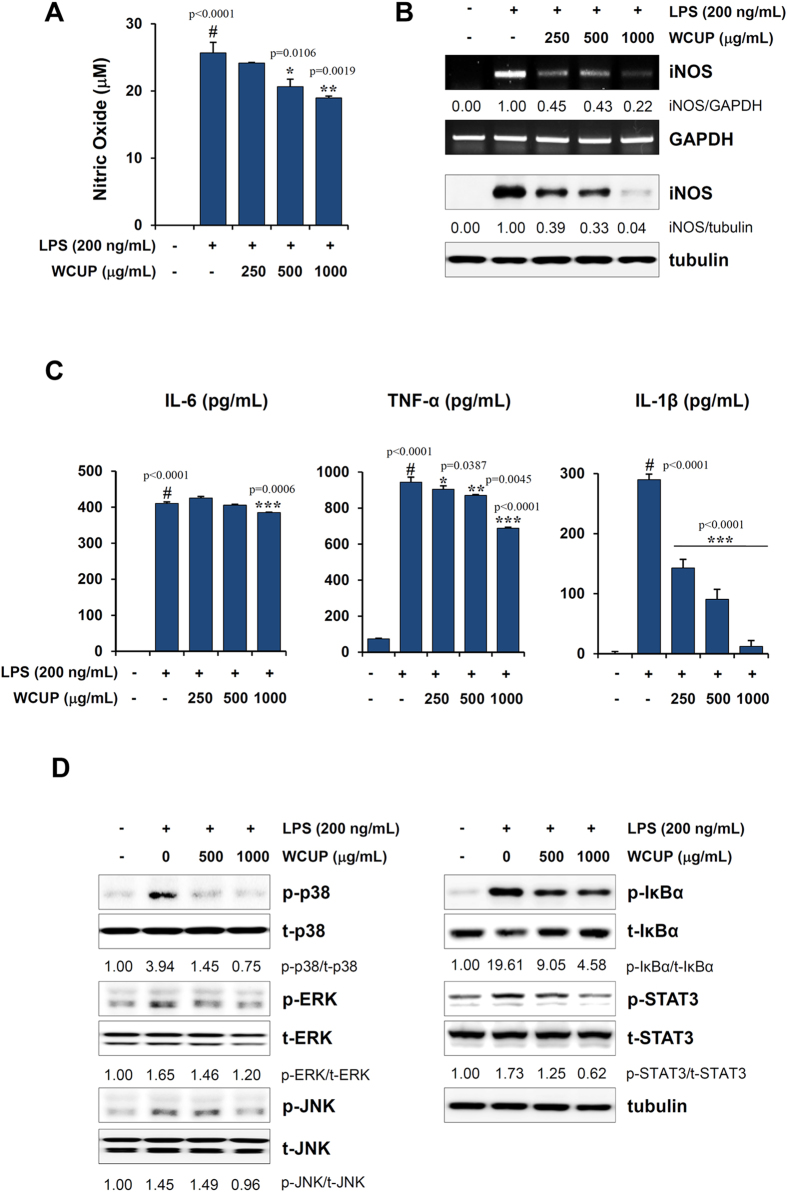
Effects of WCUP on LPS-induced production of inflammatory cytokines and MAPK/NF-κB/STAT3 activation in murine macrophage J774A.1 cells. (**A**) J774A.1 cells were pretreated with 250, 500, and 1000 μg/mL WCUP for 1 h and then stimulated with 200 ng/mL LPS for 24 h. Culture supernatants were collected and measured for NO production. The data are representative of independent experiments performed in triplicate and expressed as means ± SD. Statistical significance was evaluated with Student *t*-test. ^#^*p* < 0.001 vs. untreated control, *p < 0.05 and **p < 0.01 vs. WCUP-untreated control cells. (**B**) J774A.1 cells pretreated with the indicated concentrations of WCUP for 1 h were stimulated with 200 ng/mL LPS for 24 h. mRNA and protein levels of iNOS were examined by RT-PCR and Western blotting, respectively. Relative band intensities were calculated after normalization to GAPDH and tubulin using ImageJ software. The full size gels and blots were shown in the Supplementary Fig. S4 and band of interest is indicated with an arrow. (**C**) The levels of IL-6, TNF-α, and IL-1β in culture supernatants collected as described in (**A**) were measured by ELISA. The data are representative of independent experiments performed in triplicate and expressed as means ± SD. Statistical significance was evaluated with Student *t*-test. ^#^*p* < 0.001 vs. untreated control, *p < 0.05, **p < 0.01, and ***p < 0.001 vs. WCUP-untreated control cells. (**D**) J774A.1 cells were pretreated with 500 and 1000 μg/mL WCUP for 12 h and then stimulated with 200 ng/mL LPS for 30 min. After extracting proteins, the levels of p38, ERK, JNK, IκBα, STAT3, and their phosphorylated forms were detected by Western blotting. The band intensities relative to untreated control cells were calculated using ImageJ software after normalization to tubulin. The full size gels and blots were shown in the Supplementary Fig. S7 and band of interest is indicated with an arrow.

The chronic host inflammatory response in cancer patients elicits muscle degradation even in the face of adequate nutrition, and cytokines including IL-6 and TNF-α produced by the host immune cells in response to a tumor are major contributors to muscle wasting. In this study, we examined whether WCUP could inhibit the production of inflammatory cytokines under LPS stimulation and elucidated its underlying mechanism using murine macrophage J774A.1 cells. WCUP at 250, 500, and 1000 μg/mL suppressed LPS-induced NO production by 5.82%, 19.58%, and 26.10%, respectively, compared with that in the WCUP-untreated control (F = 30.37, p = 0.0001) (Fig. 4A). In addition, WCUP at 1000 μg/mL almost completely blocked the LPS-induced increase in iNOS expression at both the mRNA and protein levels (Fig. 4B). As shown in Supplementary Fig. S5, WCUP treatment significantly suppressed LPS-induced increase in the mRNA expressions of iNOS, IL-6, TNF-α, and IL-1β in J774A.1 cells with no apparent cytotoxicity at the concentrations evaluated (qRT-PCR, iNOS; F = 150.4, p < 0.0001, IL-6; F = 24.97, p = 0.0002, TNF-α; F = 25.67, p = 0.0002, IL-1β; F = 192.2, p < 0.0001). In addition, secreted protein levels of IL-6, TNF-α, and IL-1β in LPS-stimulated J774A.1 cells were efficiently decreased by WCUP treatment in a dose-dependent manner (IL-6; F = 75.29, p < 0.0001, TNF-α; F = 127.8, p < 0.0001, IL-1β; F = 250.1, p < 0.0001) (Fig. 4C). Since the activations of mitogen-activated protein kinase (MAPK), NF-κB, and STAT3 are involved in the production of pro-inflammatory cytokines, we examined whether these proteins are regulated by WCUP treatment. The levels of phosphorylated p38, ERK, JNK, IκBα, and STAT3 were significantly increased after LPS stimulation compared with the levels in untreated control cells, while WCUP treatment remarkably decreased the levels of phosphorylated p38, ERK, JNK, IκBα, and STAT3 (Fig. 4D). In a time course experiment, the inhibitory effects of WCUP on MAPK and NF-κB activation were demonstrated more clearly (Fig. S6). Similar to observations in J774A.1 cells, WCUP efficiently suppressed LPS-induced NO production, iNOS expression, and pro-inflammatory cytokine production in primary peritoneal macrophages (Fig. S8). In addition, WCUP did not affect the viability of peritoneal macrophages, ruling out the possibility of cytotoxic effects.

### WCUP prevents CT-26-mediated suppression of C2C12 myoblast proliferation

**Figure 5:**
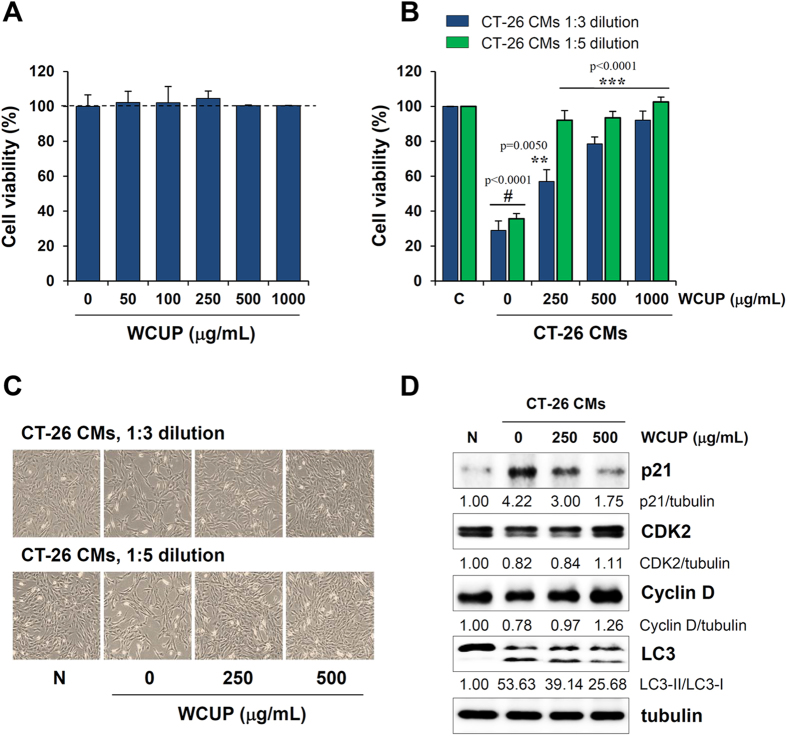
Effects of WCUP on C2C12 myoblast proliferation. **(A)** C2C12 myoblasts were treated with increasing concentrations of WCUP (50-1000 μg/mL) diluted in GM for 48 h, and then cell viability was determined using a CCK-8 kit. The data are representative of independent experiments performed in triplicate and expressed as means ± SD. **(B)** C2C12 myoblasts were incubated with WCUP-treated or -untreated CT-26 CM after 1:3 and 1:5 dilutions in GM. After 36 h of incubation, viable cells were measured using a CCK-8 kit. The data are means ± SD and representative of independent experiments performed in triplicate. Statistical significance was evaluated with Student *t*-test. ^#^*p* < 0.001 vs. GM, **p < 0.01 and ***p < 0.001 vs. WCUP-untreated CT-26 CM. **(C)** After exposure to WCUP-treated or -untreated CT-26 CM for 36 h, cells were observed under an inverted microscope. **(D)** The levels of cell cycle-related proteins were detected by Western blotting. The relative band intensities were quantitated after normalization to tubulin expression. The full size gels and blots were shown in the Supplementary Fig. S9 and band of interest is indicated with an arrow.

In cancer cachexia, tumor-derived factors including pro-inflammatory cytokines, Mstn, activin, and PIF are inextricably linked to skeletal muscle wasting^24^,^25^. In previous studies, it was demonstrated that treatment with conditioned medium (CM) derived from CT-26 cells suppressed C2C12 myoblast proliferation by regulating cell cycle-related proteins. In addition, CT-26 CM attenuated C2C12 myoblast differentiation and promoted C2C12 myotube wasting by increasing the expression of E3 ligases in myotubes such as MAFbx and MuRF-1 and by elevating intracellular protein degradation through the ubiquitin-proteasome pathway^24^. To examine the effects of WCUP on CT-26-induced muscle wasting, we first examined C2C12 myoblast proliferation after incubation in WCUP-treated or -untreated CT-26 CM at dilutions of 1:3 and 1:5 in growth medium (GM) for 36 h. WCUP itself did not affect the viability of C2C12 myoblast cells (Fig. 5A). WCUP-untreated CT-26 control CM significantly inhibited C2C12 myoblast proliferation by approximately 71.06% and 64.33% at dilutions of 1:3 and 1:5, respectively, compared with GM (1:3 and 1:5 dilution, p < 0.0001), while 250, 500, and 1000 μg/mL WCUP-treated CT-26 CM did not severely retard C2C12 myoblast proliferation (1:3 dilution; F = 77.41, p < 0.0001, 1:5 dilution; F = 186.0, p < 0.0001). Interestingly, cells in WCUP-treated CT-26 CM at 1000 μg/mL were almost completely maintained, similar to cells in GM (Fig. 5B,C). Consistent with previous studies, exposure to CT-26 control CM significantly increased p21 expression and decreased CDK2 and cyclin D expression in C2C12 myoblast cells. However, changes in these proteins after exposure to WCUP-treated CT-26 CM were insignificant, in accordance with their effects on proliferation (Fig. 5D).

### WCUP attenuates CT-26-mediated C2C12 myotube atrophy

**Figure 6:**
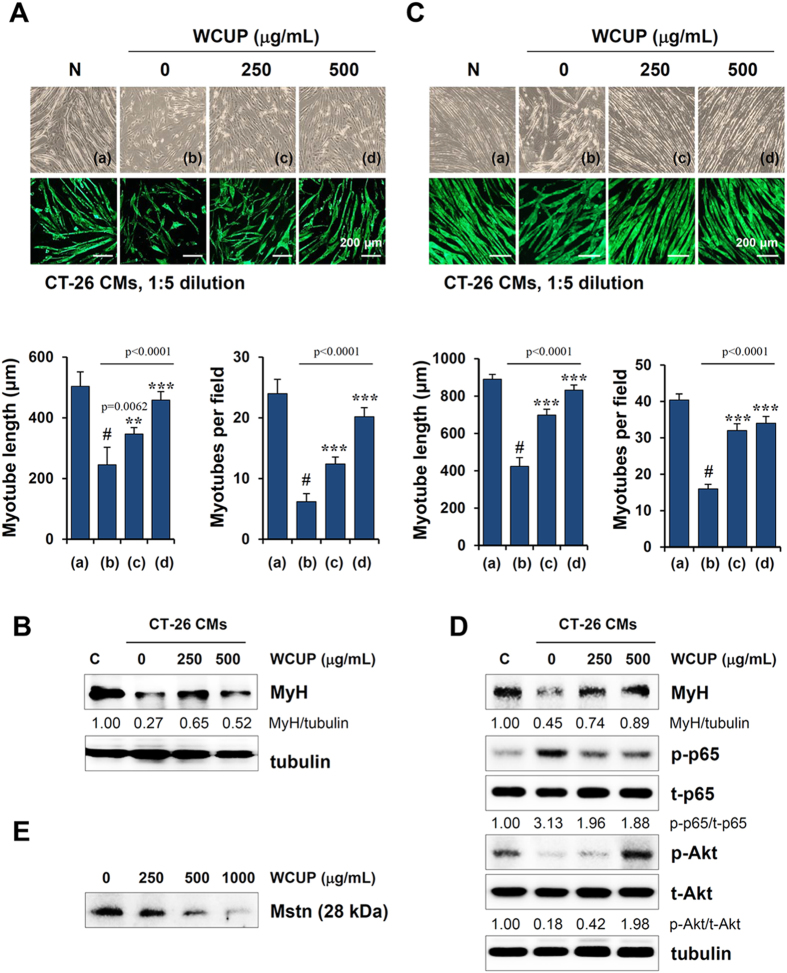
Effects of WCUP on the differentiation of C2C12 myoblasts and on the wasting of C2C12 myotubes. **(A)** C2C12 myoblasts at a density of 80% were differentiated by incubating in DM or in WCUP-treated or -untreated CT-26 CM after a 1:5 dilution in DM. After 4 days, cells were observed under an inverted microscope (upper) and detected for MyH expression by ICC (lower). Myotube length (8–10 tubes per group) and myotube number (5 fields per group) were examined and data are expressed as means ± SD. Statistical significance was evaluated with Student *t*-test. ^#^*p* < 0.001 vs. DM, **p < 0.01 and ***p < 0.001 vs. WCUP-untreated CT-26 CM. **(B)** The levels of MyH in cells were measured by Western blotting, and the relative band intensities were calculated after normalization to tubulin expression. **(C)** The C2C12 myotubes were incubated in DM or in WCUP-treated or -untreated CT-26 CM after a 1:5 dilution in DM. After 2 days, myotube degradation was observed under an inverted microscope (upper), and MyH expression was detected by ICC (upper). Myotube length (8–10 tubes per group) and myotube number (5 fields per group) were calculated and data are expressed as means ± SD. Statistical significance was evaluated with Student *t*-test. ^#^*p* < 0.001 vs. DM, ***p < 0.001 vs. WCUP-untreated CT-26 CM. **(D)** The levels of MyH, p65 phosphorylation, and Akt phosphorylation were measured by Western blotting after incubating C2C12 myotubes in DM or in WCUP-treated or -untreated CT-26 CM for 48 h. Relative band intensities were calculated after normalization to tubulin expression. **(E)** WCUP-treated or -untreated CT-26 CM were concentrated using a Centricon (Amicon Ultra centrifugal filter, 30 K; Millipore Co., Billerica, MA, USA) and then analyzed for Mstn expression by Western blotting. The full size gels and blots were shown in the Supplementary Fig. S10 and band of interest is indicated with an arrow.

We next examined the effect of WCUP on CT-26-mediated inhibition of C2C12 myoblast differentiation and CT-26-mediated C2C12 myotube wasting. WCUP-untreated control CT-26 CM diluted 1:5 in differentiation medium (DM) remarkably impaired C2C12 differentiation compared with control DM (myotube length; p < 0.0001, myotube number; p < 0.0001) whereas 250 and 500 μg/mL WCUP-treated CT-26 CM slightly suppressed it in terms of myotube length and myotube number (myotube length; F = 74.46, p < 0.0001, myotube number; F = 142.0, p < 0.0001) (Fig. 6A). Immunoblot analysis demonstrated that myosin heavy chain (MyH) expression was significantly decreased in C2C12 myoblasts differentiating in control CT-26 CM compared with DM, while WCUP remarkably prevented the CT-26-mediated decrease in MyH expression (Fig. 6B). Next, we examined whether WCUP could prevent CT-26-mediated C2C12 myotube wasting. Well-differentiated C2C12 myotubes were significantly destroyed by exposure to control CT-26 CM (1:3 dilution with DM) and exhibited the typical appearance of muscle wasting. By contrast, C2C12 myotubes incubated in WCUP-treated CT-26 CM remained almost intact, similar to DM-treated controls. ICC for MyH also clearly showed that 250 and 500 μg/mL WCUP treatment efficiently alleviated the CT-26-mediated C2C12 myotube wasting (myotube length; F = 335.0, p < 0.0001, myotube number; F = 171.8, p < 0.0001) (Fig. 6C). As demonstrated in previous studies^25^, we confirmed that CT-26 control CM decreased MyH expression and Akt phosphorylation and increased p65 phosphorylation in C2C12 myotubes. Consistent with its preventive effects on muscle wasting, WCUP significantly reversed CT-26-mediated changes in MyH, Akt phosphorylation, and p65 phosphorylation (Fig. 6D). In addition, in a dose-dependent manner in CM, WCUP significantly reduced the level of a secreted pro-cachectic factor, Mstn, in CT-26 cells (Fig. 6E).

### Identification of the main components in WCUP using HPLC

**Figure 7:**
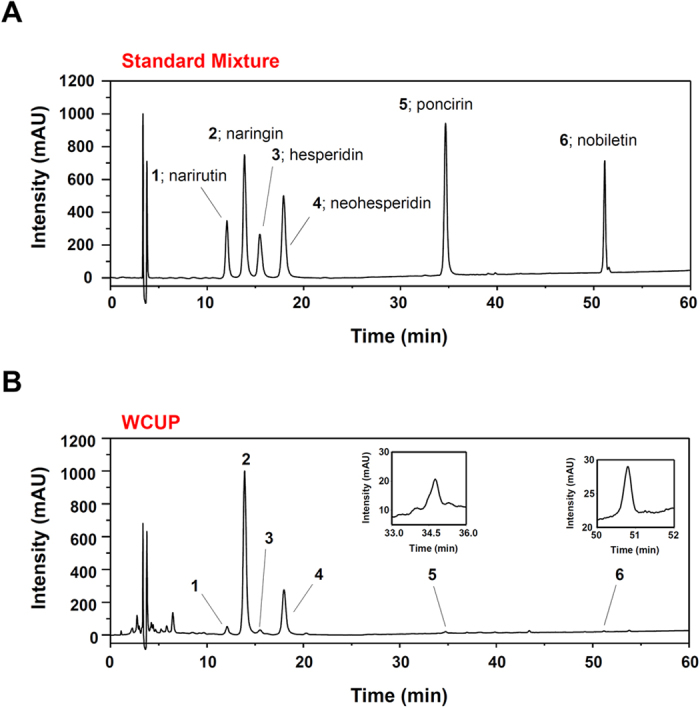
Chromatograms of six major standard compounds in WCUP. Using HPLC-DAD, six standard compounds, narirutin (1), naringin (2), hesperidin (3), neohesperidin (4), poncirin (5), and nobiletin (6) were identified in standard mixture (**A**) and in WCUP (**B**) at a wavelength of 280 nm.

Six phytochemicals in WCUP were analyzed by HPLC: narirutin, naringin, hesperidin, neohesperidin, poncirin, and nobiletin. To achieve optimal separation, gradient elution of water and acetonitrile was applied. TFA was used to inhibit peak tailing and enhance peak shape, and the UV wavelengths of these six compounds were controlled based on the maximal UV absorption of each. Each compound in the WCUP was identified by comparing the retention time (t_R_) and UV spectra of standard compounds. Narirutin (1, t_R_: 12.05 min), naringin (2, t_R_: 13.87 min), hesperidin (3, t_R_: 15.45 min), neohesperidin (4, t_R_: 17.92 min), poncirin (5, t_R_: 34.74 min), and nobiletin (6, t_R_: 34.67 min) were identified in WCUP (Fig. 7).

## Discussion

Unintentional weight loss is regarded as a medically serious condition when more than 10% of body weight is lost over a 6-month period or 5% within the last month, despite adequate nutritional intake. Continuing weight loss may deteriorate into a wasting condition called cachexia. Symptoms in patients with advanced cachexia include severe skeletal muscle loss, anorexia, early satiety, lack of energy and strength, anemia, and alterations in immune function. Cachexia is considered a critical cause of morbidity and mortality in cancer patients, occurring in up to 80–90% of patients with advanced tumors of pancreatic and gastric origin and leading to cancer-related death in 20% of cases. Loss of muscle mass is a hallmark of cancer-induced cachexia and is closely related to poor efficacy of anti-cancer therapies and more severe side effects during chemotherapy^3^. Skeletal muscle catabolism is mediated by three major proteolytic pathways, including the lysosomal system, the calcium-activated system, and the ubiquitin-proteasome pathway. In the ubiquitin-proteasome proteolytic pathway, two muscle-specific E3 ligases, MAFbx and MuRF-1, play pivotal roles in the degradation of myofibrillar and intracellular proteins, and their expression during cancer cachexia is regulated by pro-inflammatory cytokines such as TNF-α and IL-6. In addition, tumor-derived factors such as Mstn and PIF have also been implicated in the development of skeletal muscle wasting^24^,^26^,^27^. Therefore, blockade of these pro-cachectic factors effectively reduces cachexia symptoms in tumor-bearing mice.

CUP is dried skin from the Korean citrus fruit *Citrus unshiu* Markov., which belongs to the Rutaceae family and is used as a traditional herbal medicine to treat the common cold, gastrotympanites, nausea, vomiting, and dyspepsia. Previous studies reported that ethyl acetate extracts of CUP and its constituent nobiletin markedly inhibited hepatitis C virus infection in MOLT-4 cells (a human lymphoblastoid leukemia cell line)^28^. In addition, CUP strongly suppressed tumor growth in mice exposed to renal carcinoma cells by boosting cytokines such as IFN-γ and TNF-α, and it showed potent anti-tyrosinase activity in B16F10 cells as well as anti-osteoporosis activity in ovariectomized (OVX) rats^29^,^30^,^31^. Recently, it has been demonstrated that ethanol extract of CUP has the potential to ameliorate hyperglycemia and hepatic steatosis in type 2 diabetic *db/db* mice by modulating the levels of anti-inflammatory cytokines (e.g., adiponectin and IL-10) and pro-inflammatory cytokines (e.g., IL-6, monocyte chemotactic protein-1, IFN-γ, and TNF-α) in the liver and plasma^22^. Moreover, water extract of CUP inhibited pro-inflammatory cytokines in LPS-stimulated RAW 264.7 macrophages through the suppression of NF-κB activation and MAPK phosphorylation^21^.

In the current study, we found that, in CT-26 tumor-bearing mice, body weight recovered considerably by oral administration of WCUP compared with saline treatment, while tumor growth and food intake were similar between saline- and WCUP-treated mice. Skeletal muscle mass, fat mass, and hemoglobin levels were also increased by WCUP administration, suggesting beneficial effects of WCUP on cancer-induced cachexia symptoms without affecting appetite or tumor growth (Figs 1 and 2). In normal mice with no tumor, repeated administration of WCUP at doses of 250 or 500 mg/kg for 15 days did not cause significant difference in body weight (data not shown). Next, WCUP administration prevented increases in serum IL-6 levels and muscular E3 ligase levels caused by CT-26 tumor burden (Fig. 3), resulting in the recovery of skeletal muscle mass and lean body mass. In J774A.1 and peritoneal macrophages under LPS stimulation, we observed that WCUP dramatically inhibited the production of NO as well as pro-cachectic cytokines, including IL-6, TNF-α, and IL-1β, via suppression of MAPK phosphorylation and NF-κB/STAT3 activation (Fig. 4). Furthermore, WCUP decreased the CT-26 cell-derived production of IL-6 and myostatin, by which WCUP-treated CT-26 CM showed less impairment of C2C12 myoblast proliferation and differentiation and prevention of tumor-induced C2C12 myotube wasting, compared with WCUP-untreated control CT-26 CM (Figs 5 and 6). These findings indicate that WCUP suppresses the levels of humoral and tumor-derived pro-cachectic factors and efficiently prevents cancer-induced severe weight loss. Certain compounds identified from WCUP including narirutin, naringin, hesperidin, and nobiletin have been reported to exhibit potent anti-inflammatory activities^32^,^33^,^34^. In addition, hesperidin has been demonstrated to promote MyoD-induced myogenic differentiation and to accelerate injury-induced muscle regeneration in mice^35^. These results suggest that these compounds in WCUP may contribute to its anti-cachectic effects in CT-26 tumor-bearing mice. In the further study, we will identify active compounds in WCUP and elucidate the mechanisms involved in the anti-cachectic effects.

Currently, dietary supplementations, including herbal medicines, have received increasing attention as adjuvants to diminish complications in tumor-bearing mice. Treatment with fish oil and selenium prevented increases in IL-6, TNF-α, and myostatin levels in mice receiving chemotherapy with docetaxel and significantly attenuated skeletal muscle atrophy^36^. Some natural herbs and their components, including sophocarpine and matrine from *Sophora flavescens Ait*. (Kushen) and berberine and quercetin from *Rhizoma coptidis*, prevented cachexia-related symptoms in mice bearing Colon-26 adenocarcinoma^37^,^38^. In a recent study, we reported that Sosiho-tang (SO) ameliorated cachexia-related symptoms in tumor-bearing mice by retarding tumor growth and reducing systemic inflammation and muscle loss, suggesting that it may be very useful for cancer patients with severe weight loss^39^.

In summary, the present study demonstrated that WCUP reduces systemic inflammation in tumor-bearing mice and suppresses the production of pro-cachectic factors in tumors, followed by the prevention of skeletal muscle atrophy and weight loss. Identification of the active ingredients in WCUP responsible for the improvements in skeletal muscle atrophy and inflammation is now underway. Furthermore, to confirm that WCUP can act as a safe and potent anti-cancer adjuvant, its preventive/therapeutic effects against chemotherapy-induced muscle loss and its synergistic anti-cancer efficacy with chemotherapeutic agents are under investigation.

## Materials and Methods

### Cell culture and preparation of conditioned medium (CM)

CT-26 murine colon carcinoma cells (ATCC^R^ CRL-2638^TM^) and C2C12 murine myoblast cells (ATCC^R^ CRL-1772^TM^) were obtained from the American Type Culture Collection (ATCC) (Manassas, VA, USA). J774A.1 murine macrophage-like cells (KCLB no. 40067) were purchased from the Korean Cell Line Bank (Seoul, Korea). Cells were maintained in RPMI 1640 or DMEM (Lonza, Walkersville, MD, USA) supplemented with 10% (v/v) heat-inactivated fetal bovine serum (FBS; Cellgro, Manassas, VA, USA) and penicillin (100 U/mL)/streptomycin (100 μg/mL) (Cellgro) in a humidified 5% CO_2_ incubator at 37 °C. For CM collection, CT-26 cells were plated in 100-mm culture dishes at a density of 5 × 10^4^/cm^2^ and treated with or without WCUP for 24 h in complete media conditions. After washing completely, cells were incubated for 24 h in serum free DMEM and the resulting CMs were centrifuged to remove debris and filtered using a 0.22 μm disk filter. CMs were diluted with either DMEM containing 10% FBS and antibiotics (growth medium; GM) or DMEM containing 5% horse serum (HS; Gibco-BRL, Grand Island, NY, USA) and antibiotics (differentiation medium; DM) for myoblast or myotube treatment, respectively. Prior to dilution, an appropriate quantity of FBS, HS, or antibiotics were compensated. Myogenic differentiation was induced by incubating C2C12 myoblast at a density of 70-80% in DMEM containing 5% HS for 5-7 days.

### Animals

Male BALB/c mice at 6 weeks of age were obtained from Taconic Farms Inc. (Samtako Bio Korea, Osan, Korea) and maintained under specific pathogen-free (SPF) condition under 12 h light-dark cycle at 22 ± 1 °C and 55 ± 5% humidity. All animal experiments were approved by the Animal Care and Use Committee of the Korea Institute of Oriental Medicine (KIOM, Daejeon, Korea) and performed according to the guidelines of the Animal Care and Use Committee at KIOM with reference number #13–100, #14–074, and #15–011.

### Reagents and antibodies

Lipopolysaccharides (LPS) from *Escherichia coli* was obtained from Sigma Chemical Co. (St. Louis, MO, USA). Antibodies against MAFbx, MuRF1, and α-tubulin were purchased from Santa Cruz Biotechnology (Santa Cruz, CA, USA). Anti-MyH and anti-Mstn were obtained from R&D Systems (Minneapolis, MN, USA). Antibodies against iNOS, p38, p-p38 (Thr180/Tyr182), ERK, p-ERK (Thr202/Tyr204), JNK, p-JNK (Thr183/Tyr185), IκBα, p-IκBα (Ser32), STAT3, p-STAT3 (Tyr705), c-fos, p-c-fos (Ser32), c-jun, p-c-jun (Ser73), p65, p-p65 (Ser536), Akt, p-Akt (Ser473), p21, CDK2, cyclin D, and TBP were purchased from Cell Signaling Technology (Danvers, MA, USA). Anti-LC3 antibody was obtained from Sigma Chemical Co., and horseradish peroxidase (HRP)-conjugated anti-mouse and anti-rabbit antibodies were obtained from Cell Signaling Technology.

### Preparation of water extract of CUP (WCUP)

The identity of CUP obtained from Yeongcheon Oriental Herbal Market (Yeongcheon, Korea) was first confirmed by Professor Ki Hwan Bae (College of Pharmacy, Chungnam National University, Daejeon, Korea) and stored in the KIOM herbal bank. To prepare WCUP, dried CUP (50 g) was soaked in distilled water (1000 mL) and then heat-extracted at 115 °C for 3 h in a Cosmos-600 Extractor (Gyeonseo Co., Incheon, Korea). After filtration through standard testing sieves (150 μm, Retsch, Haan, Germany), WCUP was freeze-dried and kept in desiccators at 4 °C. The amount of WCUP powder collected was 11.89 g, and the yield was 23.78%. For *in vitro* experiments, WCUP powder was dissolved in 10% DMSO to a final concentration of 50 mg/mL, filtered through a 0.22-μm disk filter, and then stored at −20 °C until use.

### Mouse cancer cachexia model

To induce cancer-mediated cachexia, CT-26 cells (2 × 10^6^ per mouse) were subcutaneously inoculated into the abdominal region of 7-week-old male BALB/c mice. Body weight, tumor volume, and food intake were measured twice a week during the experiment. Food intake was calculated as the mean value of five mice per cage. On day 10 after tumor inoculation, when significant decreases in body weight and food intake were observed in tumor-bearing mice, the mice were divided randomly into three groups and orally administered saline or WCUP daily at doses of 250 and 500 mg/kg. WCUP was suspended in the saline and administered orally in a volume of 100 μL using disposable animal feeding needles purchased from Fuchigami (Kyoto, Japan). Age-matched healthy control mice having no tumors were treated with an equal volume of saline. On day 27 after tumor inoculation, the mice were sacrificed by intraperitoneal injection with a 2:1 mixture of Zoletil (Virbac, Magny-en-Vexin, France) and Rumpun (Bayer, Seoul, Korea) (200 μL per mouse). The tumor, heart, gastrocnemius muscle, and epididymal fat were resected, and blood samples were collected. Whole-blood and serum samples were examined for hemoglobin (Hb) levels using the ADVIA 2120i hematology system (Siemens Healthcare Diagnostics, Tarrytown, NY, USA) and IL-6 levels using the ELISA antibody kit (eBioscience, San Jose, CA, USA), respectively. For measurement of carcass weight, the remaining viscera and blood were completely removed and clearly wiped out with gauze pad.

### Reverse transcription and polymerase chain reaction (RT-PCR)

Total RNA was extracted using RNA extraction solution (BioAssay Co., Daejeon, Korea) according to the manufacturer’s instruction. RNA concentrations were measured using a NanoDrop ND-1000 spectrophotometer (NanoDrop Technologies, Wilmington, DE) and 3 μg RNA was reverse transcribed using 1^st^ Strand cDNA Synthesis kit (BioAssay Co.). Quantitative real-time PCR was performed using QuantStudio 6 Flex Real-Time PCR system (Thermo Scientific, Rockford, IL, USA) as described previously^40^. For each gene, the *Ct* values were normalized to those of β-actin and then presented as fold differences compared to untreated control cells. cDNA aliquots were also analyzed by semi-quantitative PCR as described previously^21^, and the PCR products were visualized by electrophoresis on 1% agarose gels and staining with GreenLight^TM^ (BioAssay Co.). The band intensity was measured using ImageJ software (National Institute of Health, USA).

### Western blot analysis

Whole cell lysates and nuclear fractions were obtained using M-PER Mammalian Protein Extraction Reagent and NE-PER Nuclear and Cytoplasmic Extraction Reagent (Thermo Scientific, Rockford, IL), respectively. Proteins were separated using SDS-PAGE and immunoblotted using specified primary antibodies and HRP-conjugated secondary antibodies as described previously^41^. Target proteins were visualized using Bio-Rad Clarity^TM^ Western ECL Substrate and ChemiDoc^TM^ Touch Imaging System (Bio-Rad, Hercules, CA, USA). Relative band intensity was measured using ImageJ software.

### Detection of NO levels

Cells pre-treated with indicated concentrations of WCUP for 1 h were stimulated with 200 ng/mL LPS for 24 h. Culture supernatant was collected and mixed with an equal volume of Griess reagent (1% sulfanilamide, 0.1% naphthylethylenediamine dihydrochloride, and 2.5% phosphoric acid). After incubation for 5 min at RT, the absorbance was measured at 570 nm using SpectraMaxi3 Multi-mode reader (Molecular Devices, Sunnyvale, CA, USA).

### Preparation of murine peritoneal macrophages and stimulation condition

Male ICR mice were intraperitoneally injected with sterile 3% sodium thioglycollate (Sigma Chemical Co., St. Louis, MO, USA) in a volume of 300 μL per mouse. After 3 days, mice were sacrificed by CO_2_ inhalation and macrophages were collected by washing peritoneal cavity with 10 mL of ice-cold PBS. After RBC lysis, cells suspended in 10% FBS/RPMI medium were incubated in a humidified 5% CO_2_ incubator at 37 °C overnight to attach into the surface of culture plates. For the stimulation, medium was replaced with fresh one and then LPS (200 ng/mL) was added in the presence or absence of WCUP.

### Enzyme-linked immunosorbent assay (ELISA)

The levels of murine IL-6, TNF-α, and IL-1β in the serums and culture supernatants were determined using ELISA antibody kits (eBioscience, San Diego, CA, USA) according to the manufacturer’s instruction.

### Cell proliferation assay

Cells were plated in a 96-well culture plate at a density of 0.5–2 × 10^4^ cells/well, treated with or without indicated concentrations of WCUP or WUP-treated or –untreated CT-26 CMs, and then incubated in a 5% CO_2_ incubator at 37 °C. At indicated time point, cell proliferation was determined using the Cell Counting Kit-8 (CCK-8; Dojindo Laboratories, Kumamoto, Japan) according to the manufacturer’s protocol.

### Immunocytochemistry (ICC)

C2C12 myoblasts or myotubes in 35-mm glass-bottomed dishes were exposed to WCUP-treated or -untreated CT-26 CM for the indicated periods. After washing with PBS, cells were fixed with 4% paraformaldehyde (PFA) in PBS (v/v) for 30 min at 25 °C and then permeabilized and blocked with ABS buffer (1 M Tris base, 1.5 M NaCl, pH 7.5) containing 0.1% Triton X-100 and 3% goat serum for 30 min at 25 °C. The expression of MyH was detected using anti-MyH antibody (diluted 1:500 in ABS buffer) overnight at 4 °C, followed by Alexa Fluor 488-goat anti-mouse IgG antibody (1:1000; Life Technologies, Eugene, OR, USA) for 3 h at 25 °C. Cells were visualized under a fluorescence microscope using CellSense Entry software (Olympus TH4-200; Olympus Optical Co Ltd, Tokyo, Japan).

### Chromatographic analysis of WCUP

The phytochemical profile of WCUP was analyzed using the Hitachi HPLC system and EZchrom Elite software (Lachrom Elite; Hitachi High-Technologies Co., Tokyo, Japan). Chromatographic separation was achieved using an OptimaPak C18 column (4.6 × 250 mm, 5 μm; RS Tech Co., Daejeon, Korea). Gradient elution using deionized water (A) and acetonitrile (B) was performed as follows: 0-20 min with 18% B and 20-60 min with 55% B. The flow rate and injection volume were 1 mL/min and 10 μL, respectively, and HPLC chromatograms were obtained using UV at 190-400 nm. Standard samples including narirutin (provided by Professor Young Ho Kim, College of Pharmacy, Chungnam National University), naringin (Sigma-Aldrich Co.), hesperidin (KFDA, Osong, Korea), neohesperidin (Sigma-Aldrich Co.), poncirin (KFDA), and nobiletin (provided by Professor Young Ho Kim) were dissolved and diluted in methanol at 0.15-200 μg/mL, and the WCUP sample was dissolved in methanol at 5 mg/mL.

### Statistics

Data were expressed as means ± standard deviation (SD). Statistical significance of mean values in two groups or treatment effects were analyzed with Student’s *t*-test or one-way ANOVA by Dunnett’s test using GraphPad PRISM software (GraphPad PRISM software Inc., Version 6.07, CA, USA), respectively. A *p*-value less than 0.05 was considered as significant.

## Additional Information

**How to cite this article**: Kim, A. *et al. Citrus unshiu* peel extract alleviates cancer-induced weight loss in mice bearing CT-26 adenocarcinoma. *Sci. Rep.* **6**, 24214; doi: 10.1038/srep24214 (2016).

## Supplementary Material

Supplementary Information
