# Supplementary material for: Citrus unshiu peel extract alleviates cancer-induced weight loss in mice bearing CT-26 adenocarcinoma
Source: Sci Rep. 2016 Apr 11;6:24214. doi: 10.1038/srep24214 (PMC4827095; doi:10.1038/srep24214)
Supplement: Supplementary Information [file srep24214-s1.pdf]

Supplementary Information

Citrus unshiu peel extract alleviates cancer-induced weight loss in mice bearing CT-26 adenocarcinoma

Aeyung Kim, Minju Im, Min Jung Gu, and Jin Yeul Ma*

- Supplementary Figure S1
- Supplementary Figure S2
- Supplementary Figure S3
- Supplementary Figure S4
- Supplementary Figure S5
- Supplementary Figure S6
- Supplementary Figure S7
- Supplementary Figure S8
- Supplementary Figure S9
- Supplementary Figure S10

*Correspondence and requests for materials should be addressed to J.Y.M (jyma@kiom.re.kr)

Figure S1

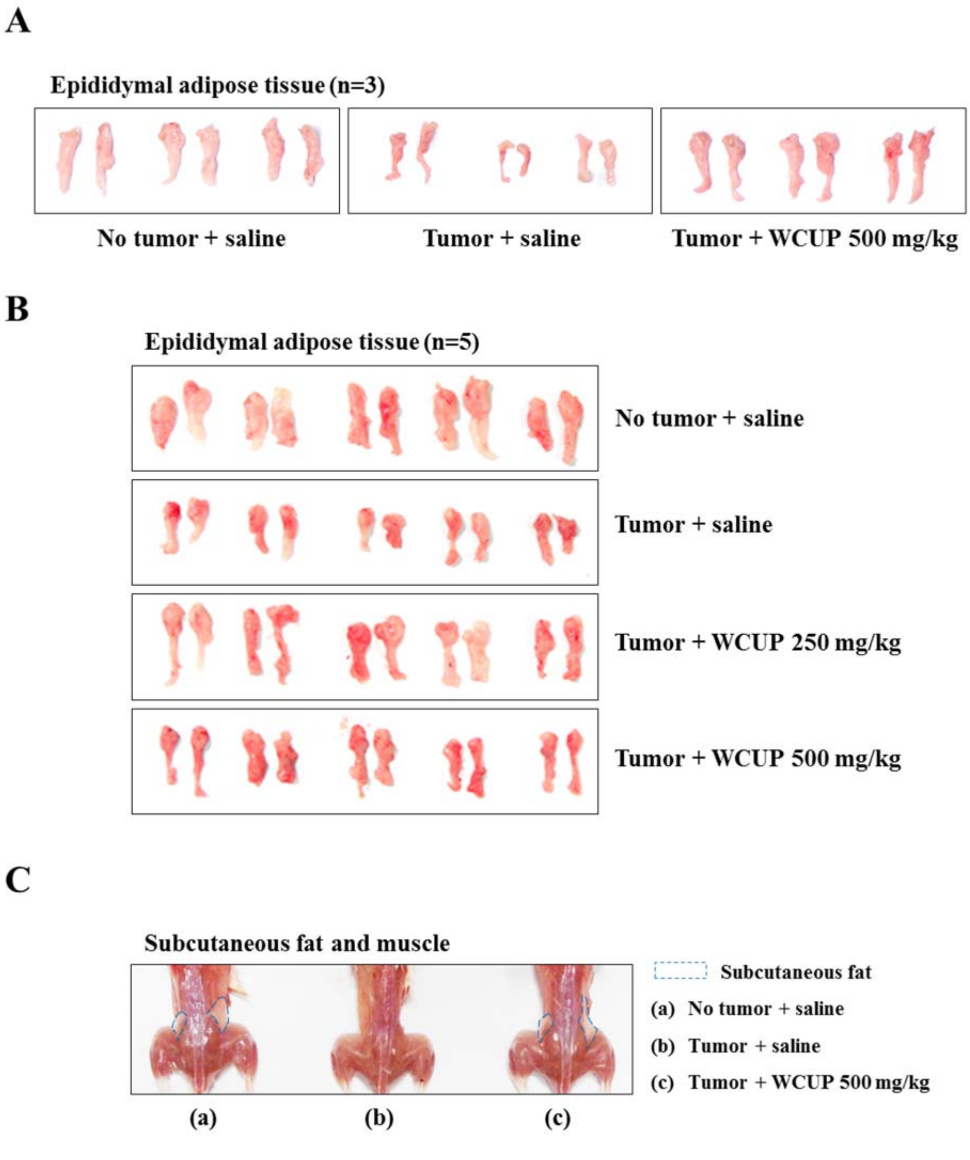

Figure S1. Effects of WCUP on the CT-26 tumor-induced cachexia. Male BALB/c mice were inoculated subcutaneously with CT-26 cells, and on day 10 after tumor inoculation, the mice were orally administered WCUP or saline daily during the experiment. Normal mice with no tumors were also treated with an equal volume of saline. **(A and B)** After mice were sacrificed, periepididymal adipose tissue was isolated and photographed. **(C)** After the mice were sacrificed, subcutaneous fat on the flank and whole body muscle were also observed.

Figure S2

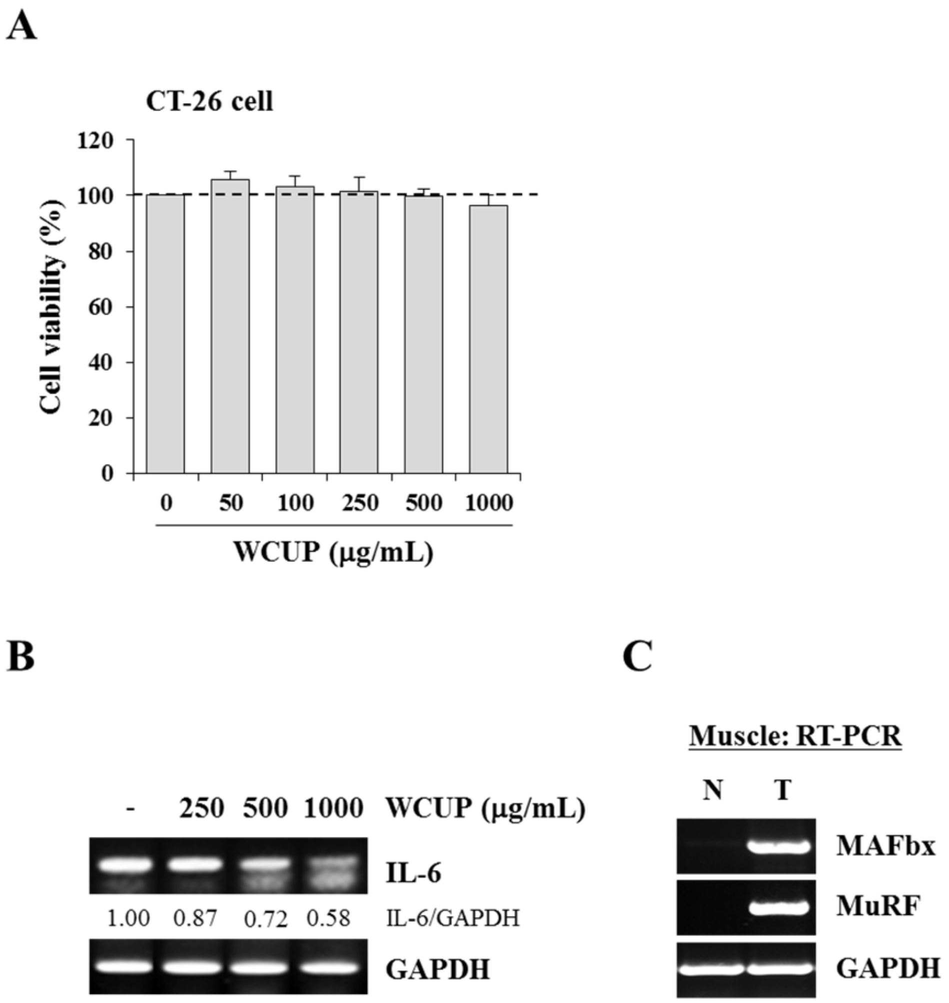

Figure S2. WCUP decreased IL-6 mRNA expression without cytotoxicity. (A) CT-26 cells were treated with the indicated concentrations of WCUP for 48 h, and cell viability was then determined using a CCK-8 kit. Data are presented as means ± SD of triplicate assays. **(B)** mRNA levels of IL-6 in CT-26 cells after WCUP treatment for 24 h were measured by RT-PCR, and the band intensities relative to those of the untreated control cells were calculated after normalization to GAPDH expression. **(C)** mRNA levels of MAFbx and MuRF-1 in gastrocnemius muscle were examined by RT-PCR. N, normal mice; T, tumor-bearing mice.

Figure S3

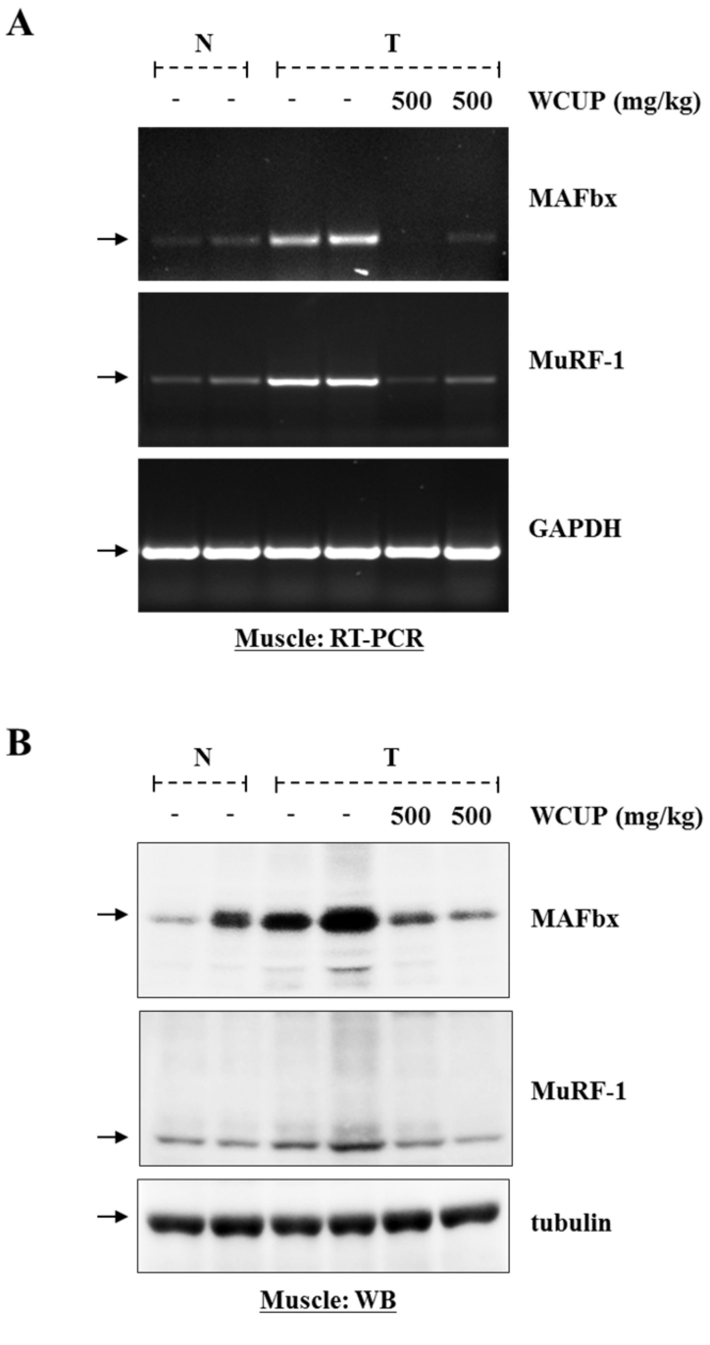

Figure S3. mRNA and protein levels of MAFbx and MuRF-1 in gastrocnemius muscle were examined by RT-PCR and Western blotting. This is a full length image of the cropped gels and blots presented in the Figure 3C and 3D.

Figure S4

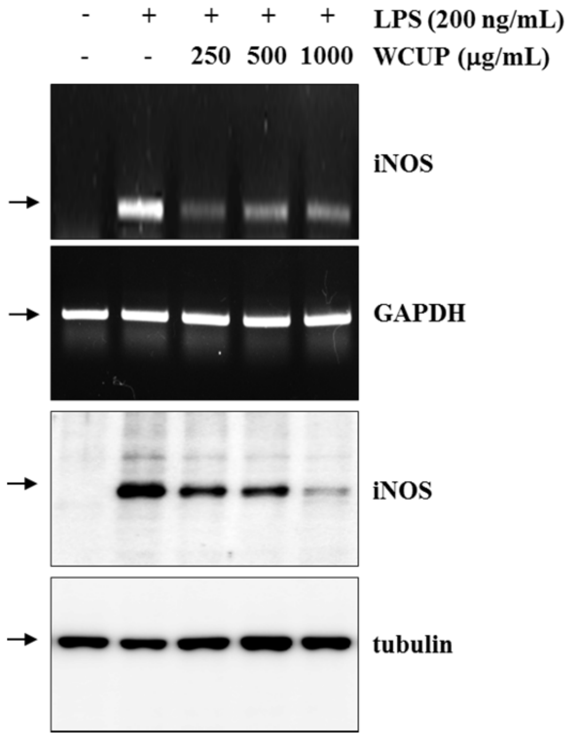

Figure S4. mRNA and protein levels of iNOS in J774A.1 cells were examined by RT-PCR and Western blotting. This is a full length image of the cropped gels and blots presented in the Figure 4B.

Figure S5

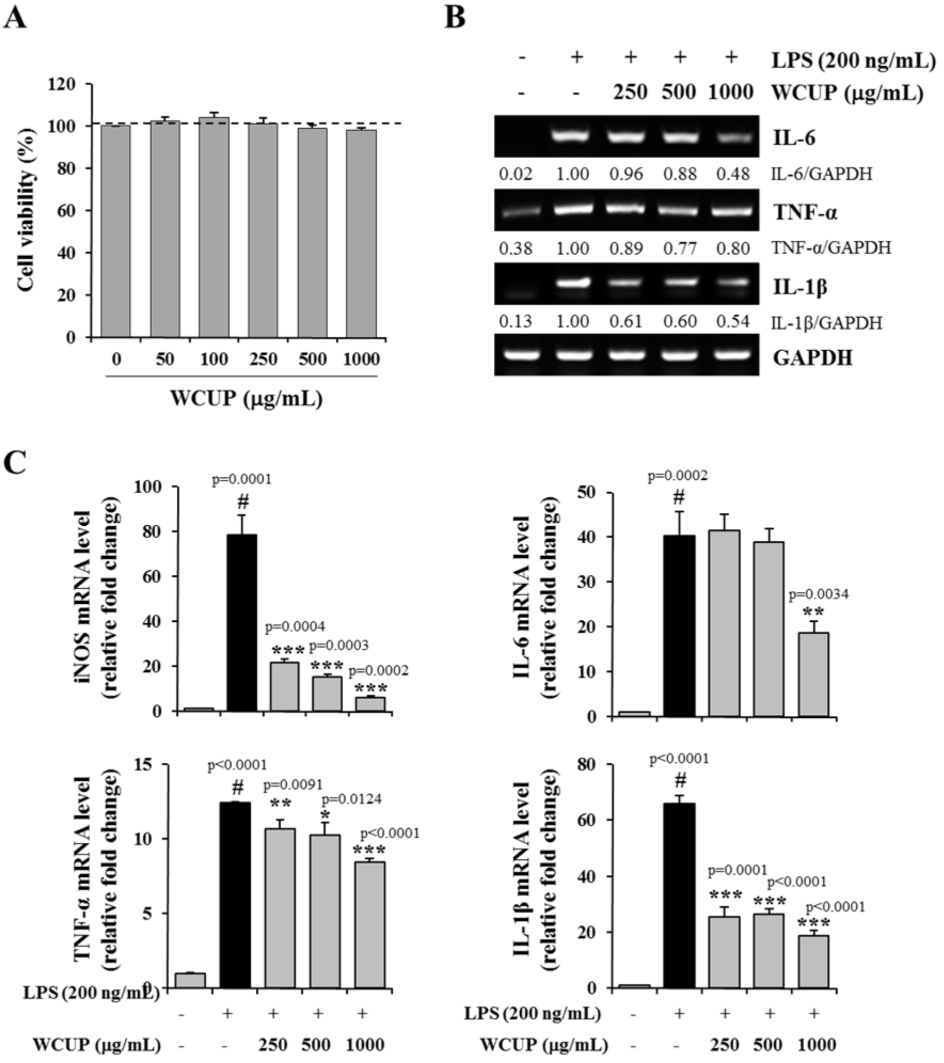

Figure S5. Effects of WCUP on the mRNA levels of inflammatory cytokines in J774A.1. (A) J774A.1 cells were treated with the indicated concentrations of WCUP for 48 h, and then cell viability was examined using a CCK-8 kit. Data are presented as means ± SD of triplicate assays. **(B)** The mRNA levels of IL-6, TNF-α, and IL-1β were determined by semi-quantitative RT-PCR. Band intensities relative to WCUP-untreated control cells were calculated after normalization to GAPDH. **(C)** The mRNA levels of iNOS, IL-6, TNF-α, and IL-1β were also analyzed by quantitative real-time RT-PCR. The level of each mRNA was presented as relative fold change compared to untreated control cells after normalization with that of β-actin. Data are presented as means ± SD of triplicate samples. Statistical significance was evaluated with Student *t*-test. #$p < 0.001$ vs. untreated control, *$p < 0.05$, **$p < 0.01$, ***$p < 0.001$ vs. WCUP-untreated control cells.

Figure S6

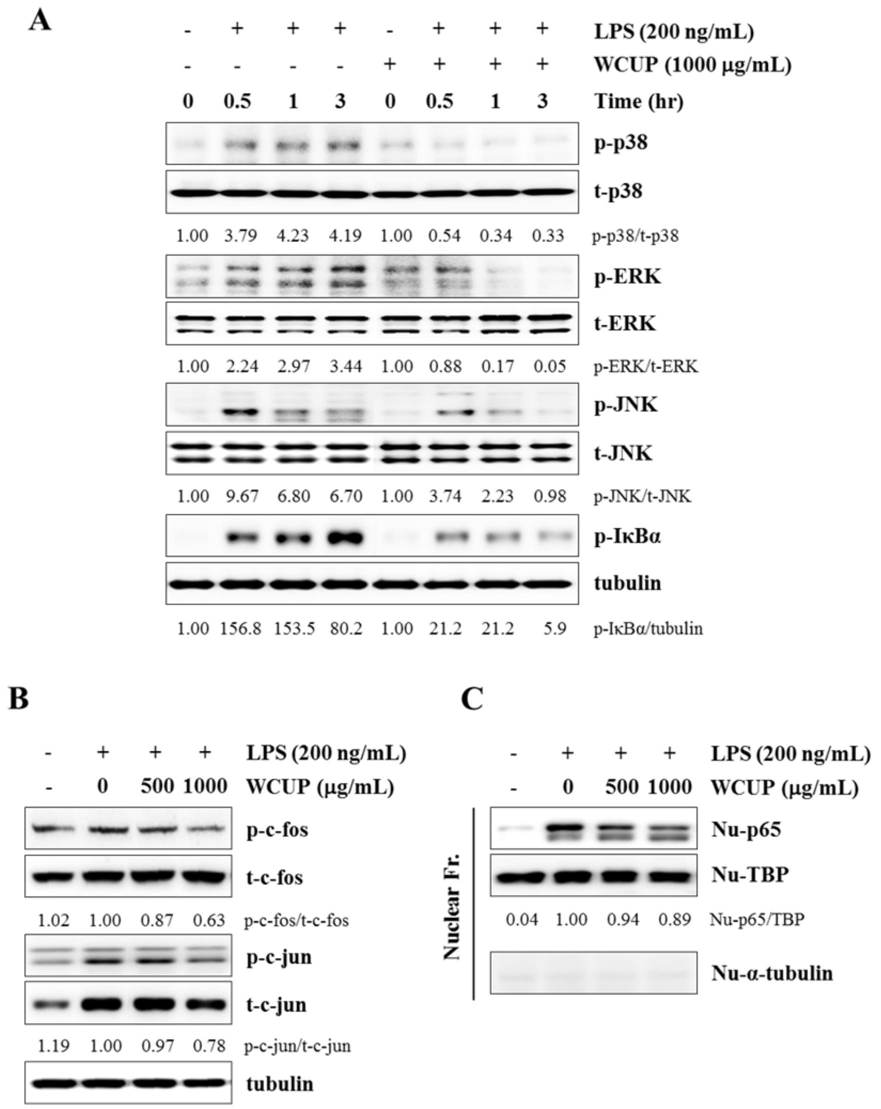

Figure S6. Effects of WCUP on LPS-induced MAPK and NF-κB activation. (A) J774A.1 cells were pretreated with 1000 μg/mL WCUP for 12 h and then stimulated with 200 ng/mL LPS for the indicated time periods. The protein levels were detected by Western blotting, and the band intensities relative to LPS-unstimulated cells were calculated after normalization to tubulin expression. **(B-C)** J774A.1 cells pretreated with the indicated concentrations of WCUP were stimulated with LPS for 30 min. c-fos and c-jun phosphorylation (B) and nuclear p65 translocation (C) were detected. Band intensities were determined after normalization to tubulin or TBP expression.

Figure S7

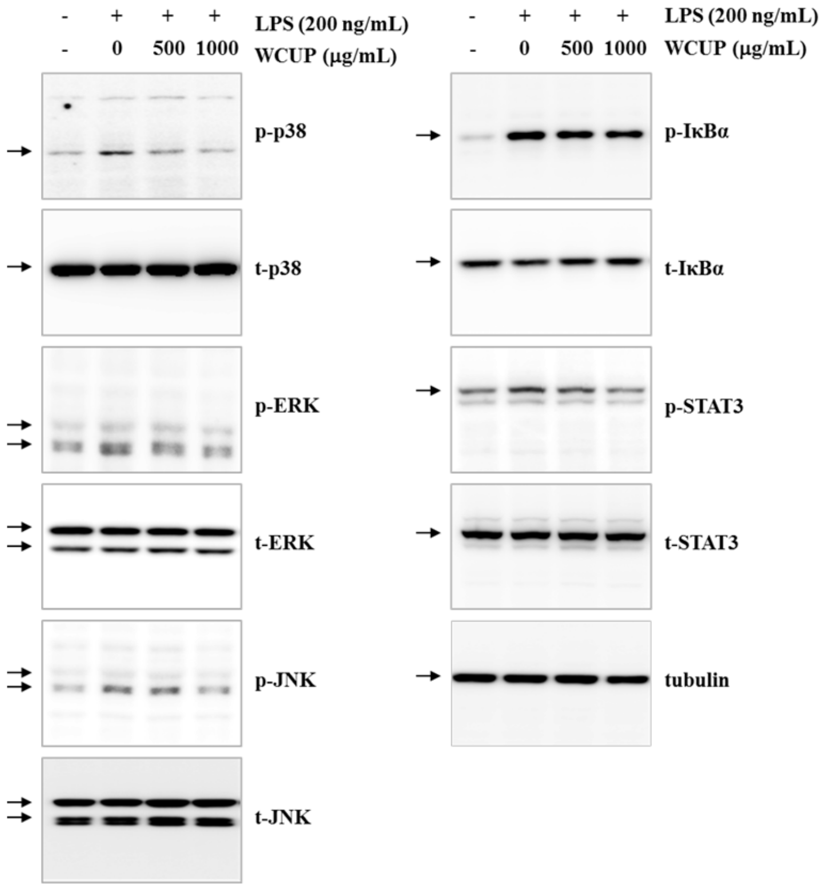

Figure S7. The levels of p38, ERK, JNK, IκBα, STAT3, and its phosphorylated forms in J774A.1 cells examined by Western blotting. This is a full length image of the blots presented in the Figure 4D.

Figure S8

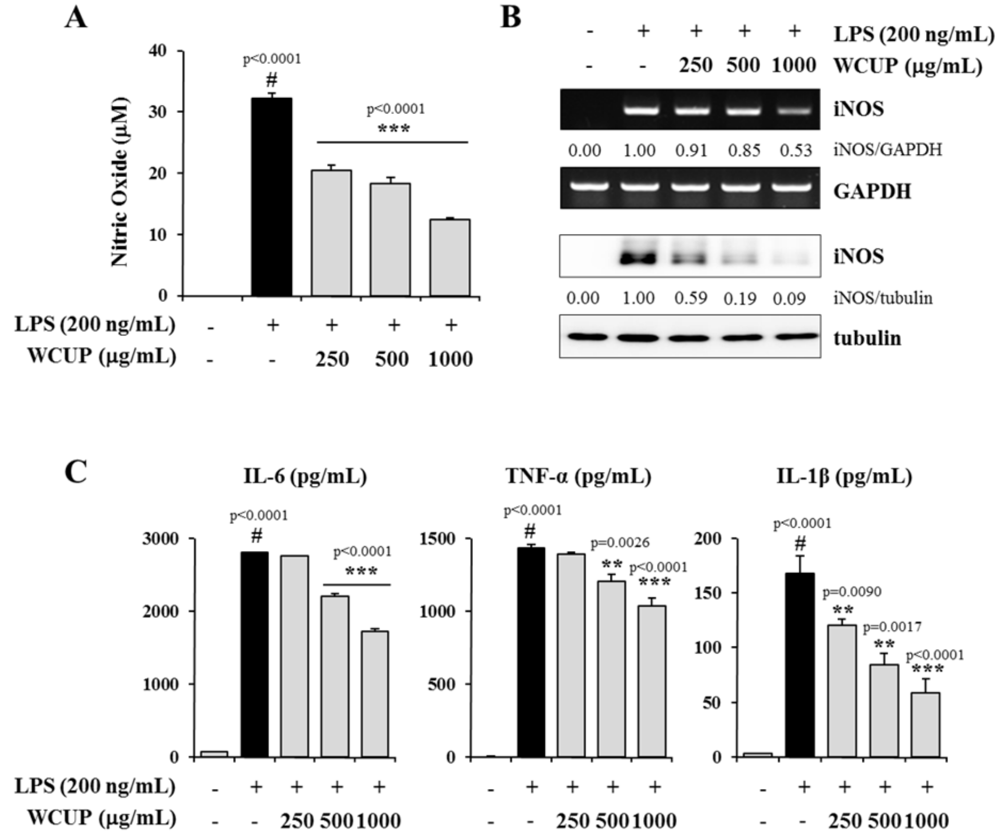

Figure S8. Effects of WCUP on LPS-induced NO and inflammatory cytokines in peritoneal macrophages. Peritoneal microphages were harvested from male ICR mice as described in Materials and methods, pretreated with or without the indicated concentrations of WCUP for 1 h, and then stimulated with LPS for 24 h. **(A)** NO levels in culture supernatants were measured. The data are representative of independent experiments performed in triplicate and expressed as means ± SD. Statistical significance was evaluated with Student *t*-test. #$p < 0.001$ vs. untreated control, ***$p < 0.001$ vs. WCUP-untreated control cells. **(B)** mRNA and protein levels of iNOS were measured by RT-PCR and Western blotting, respectively. Band intensities relative to WCUP-untreated control cells were determined after normalization to GAPDH and tubulin expression. **(C)** Levels of IL-6, TNF-α, and IL-1β in culture supernatants were measured by ELISA. The data are representative of independent experiments performed in triplicate and expressed as means ± SD. Statistical significance was evaluated with Student *t*-test. #$p < 0.001$ vs. untreated control, **$p < 0.01$, and ***$p < 0.001$ vs. WCUP-untreated control cells.

Figure S9

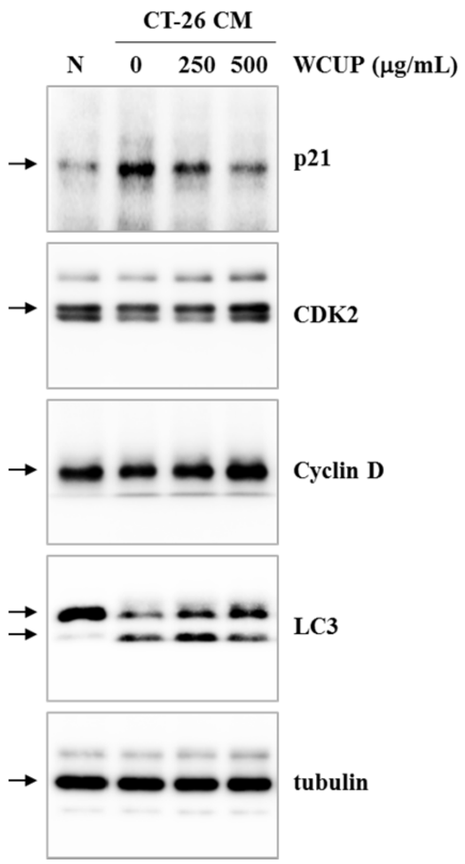

Figure S9. Cell cycle-related proteins in C2C12 cells were detected by Western blotting. This is a full length image of the blots presented in the Figure 5D.

Figure S10

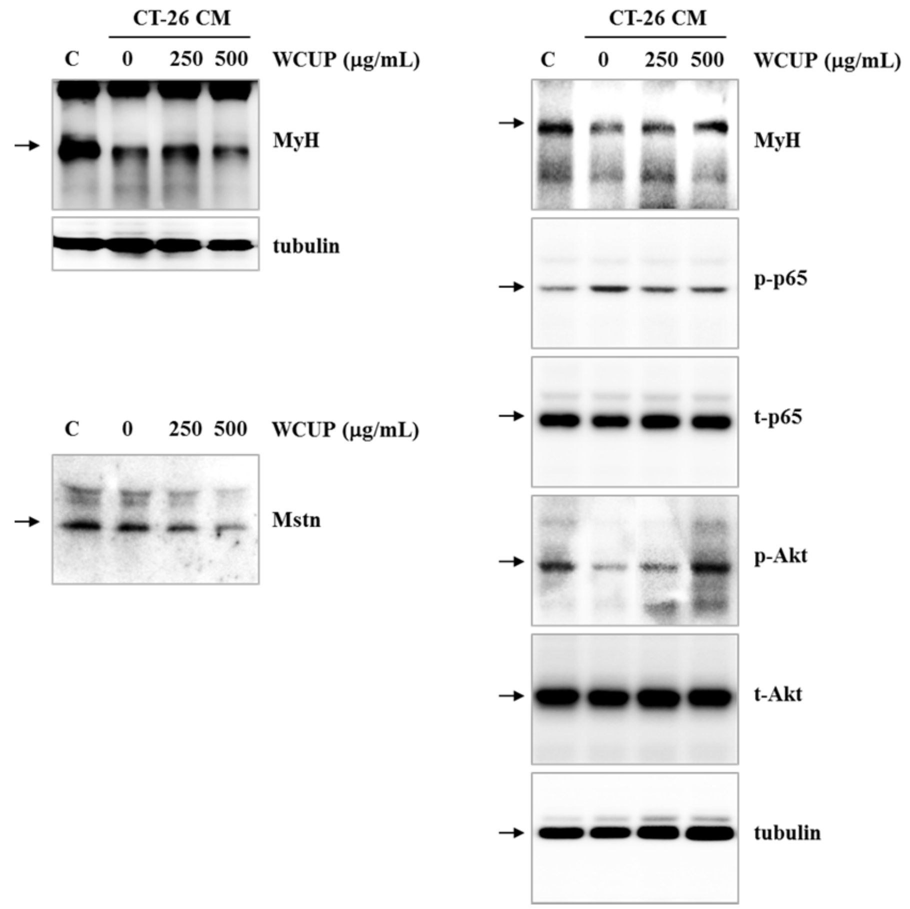

Figure S10. The levels of MyH in C2C12 myoblast, muscle wasting-related proteins in C2C12 myotubes, and Mstn in CT-26 CMs were examined by Western blotting. This is a full length image of the blots presented in the Figure 6B, 6D, and 6E.
